# Decreased expression of Rev-Erbα in the epileptic foci of temporal lobe epilepsy and activation of Rev-Erbα have anti-inflammatory and neuroprotective effects in the pilocarpine model

**DOI:** 10.1186/s12974-020-1718-7

**Published:** 2020-01-31

**Authors:** Jiong Yue, Jiaojiang He, Yujia Wei, Kaifeng Shen, Kefu Wu, Xiaolin Yang, Shiyong Liu, Chunqing Zhang, Hui Yang

**Affiliations:** 1 Department of Neurosurgery, Xinqiao Hospital, Army Medical University (Third Military Medical University), 183 Xinqiao Main Street, Shapingba District, Chongqing, 400037 China; 2 Department of Neurosurgery, West China Hospital of Sichuan University, Chengdu, China

**Keywords:** Temporal lobe epilepsy, Rev-Erbα, NLRP3 inflammasome, Neuroinflammation, Neuronal apoptosis

## Abstract

**Background:**

A hallmark of temporal lobe epilepsy (TLE) is brain inflammation accompanied by neuronal demise. Accumulating evidence demonstrates that Rev-Erbα is involved in regulating neuroinflammation and determining the fate of neurons. Therefore, we studied the expression and cellular distribution of Rev-Erbα in the epileptogenic zone of TLE and the effect of treatment with the Rev-Erbα specific agonist SR9009 in the pilocarpine model.

**Methods:**

The expression pattern of Rev-Erbα was investigated by western blotting, immunohistochemistry, and immunofluorescence labeling in patients with TLE. Next, the effects of SR9009 on neuroinflammation, neuronal apoptosis, and neuronal loss in the mouse hippocampus 7 days after status epilepticus (SE) were assessed by western blotting, immunofluorescence labeling staining, and TUNEL staining.

**Results:**

The western blotting, immunohistochemistry, and immunofluorescence labeling results revealed that Rev-Erbα was downregulated in the epileptogenic zone of TLE patients and mainly localized in neurons, astrocytes, and presumably microglia. Meanwhile, the expression of Rev-Erbα was decreased in the hippocampus and temporal neocortex of mice treated with pilocarpine in the early post-SE and chronic phases. Interestingly, the expression of Rev-Erbα in the normal hippocampus showed a 24-h rhythm; however, the rhythmicity was disturbed in the early phase after SE, and this disturbance was still present in epileptic animals. Our further findings revealed that treatment with SR9009 inhibited NLRP3 inflammasome activation, inflammatory cytokine (IL-1β, IL-18, IL-6, and TNF-α) production, astrocytosis, microgliosis, and neuronal damage in the hippocampus after SE.

**Conclusions:**

Taken together, these results suggested that a decrease in Rev-Erbα in the epileptogenic zone may contribute to the process of TLE and that the activation of Rev-Erbα may have anti-inflammatory and neuroprotective effects.

## Background

Temporal lobe epilepsy (TLE) is the most common epileptic syndrome in adults. Spontaneous recurrent seizures are characteristic of patients and animals with TLE [1, 2]. These recurrent seizures trigger a series of cellular and molecular events, which ultimately lead to the progression of epilepsy. Known critical events include astrocytosis accompanied by the activation of brain microglia and the production of inflammatory cytokines [3–6]. Subsequent neuronal apoptosis and damage gradually lead to the pathological sequelae of specific epileptogenic foci [5, 7]. Therefore, it is profitable to actively explore the underlying molecular mechanisms of these critical events.

Rev-Erbα, a circadian nuclear receptor encoded by the *NR1D1* (nuclear receptor subfamily 1, group D, member 1) gene, is a dominant transcriptional regulatory factor that represses the expression of genes that are involved in various physiological and pathophysiological processes, such as inflammation [8, 9], circadian rhythm [10], neurogenesis [11], and metabolism [8, 12], making it a potential therapeutic target for epilepsies, neurodegenerative diseases, inflammatory diseases, and metabolic disorders. For instance, targeting Rev-Erbα may represent a promising approach for the prevention and management of colitis since Rev-Erbα regulates colonic inflammation through its repressive effect on the NF-κB/NOD-like receptor family pyrin domain containing 3 (NLRP3) inflammasome axis [13]. A number of studies have shown that Rev-Erbα suppresses the expression of some pro-inflammatory cytokines and chemokines, following the binding of the Rev-Erbα-specific agonist SR9009 in macrophages [14, 15]. In the central nervous system (CNS), Rev-Erbα deletion induces spontaneous hippocampal microgliosis and cell nonautonomous astrocyte activation [16]. Further in vivo and in vitro experiments have confirmed that the deletion of Rev-Erbα aggravates lipopolysaccharide (LPS)-induced hippocampal neuroinflammation, while the pharmacological activation of Rev-Erbα can alleviate this inflammatory reaction [16]. Moreover, the stimulation of REV-Erbα regulates the tumor necrosis factor (TNF)- or LPS-induced expression of proinflammatory molecules (interleukin-1β (IL-1β), interleukin-6 (IL-6), and matrix metalloprotease-9 (MMP-9)) in astrocytes or microglia [16–18]. In addition, it is worth noting that Rev-Erbα is closely related to neuronal apoptosis and neurodegeneration in the study of Parkinson’s disease and cerebellar development [19, 20]. However, little is known about the role of Rev-Erbα in TLE.

Considering the critical role of Rev-Erbα in regulating inflammation and neuronal fate in the CNS, Rev-Erbα may be involved in pathophysiological changes in TLE. Hence, the objective of this study was to evaluate the expression patterns of Rev-Erbα in the epileptogenic foci of TLE patients and pilocarpine-treated mice. Furthermore, we detected the effects of the potent Rev-Erbα-specific agonist SR9009 on inflammatory reactions, glial proliferation, neuronal apoptosis, and neuronal loss after an episode of pilocarpine-induced SE.

## Methods

### Patient tissue collection and clinical data

Epileptic temporal neocortex samples from 22 patients with drug refractory TLE were examined. The diagnosis of each drug resistant epilepsy patient was based on the criteria established by the International League Against Epilepsy (ILAE). No other nervous system diseases (glioma, cavernous hemangioma, cyst, cortical dysplasia, etc.) were detected in these patients by brain computerized tomography (CT), high-resolution magnetic resonance imaging (MRI), long-term video-EEG monitoring, neuropsychological tests, and pathological examinations. For the control experiments, we used apparently normal temporal neocortices from ten patients being treated for increased intracranial pressure due to severe traumatic brain injury. In particular, we selected tissues from areas distant from the direct injury area and obtained < 4 h after the brain injury [21]. These subjects had no history of epilepsy or exposure to antiepileptic drugs and no notable signs of any other neurological diseases. All temporal neocortex specimens were normal, as confirmed by neuropathological examination. Relevant clinical findings of both TLE patients and control subjects can be found in (Tables 1 and 2). The age and gender did not differ between the control subjects and epilepsy patients. Studies involving human tissue were approved by the ethics committee of the Army Medical University, China. And the human brain specimens were used in a manner compliant with the Declaration of Helsinki. All patients signed informed consent for using the biologic material.

**Table 1:** Clinical characteristics of the patients with TLE

Case no.	Gender	Age (year)	Duration (year)	AEDs before surgery	Side focus	Seizure type	PO	Application
1	M	23	5	OXC, CBZ, VPA	RTN	FIAS	I	WB, IHC
2	M	18	5	OXC,VPA, PHT	LTN	FIAS	I	WB, IHC
3	M	24	9	OXC, CBZ, VPA, LTG	LTN	FAS/FBTCS	III	WB, IHC
4	F	29	14	CBZ, VPA, CBL	LTN	FAS/FBTCS	I	WB, IHC
5	F	31	12	OXC, CBL, VPA	LTN	FIAS	II	WB, IHC
6	M	19	5	OXC, VPA, GBP	RTN	FIAS	I	WB, IHC
7	M	31	10	CBZ, VPA, TPM	LTN	FIAS/FBTCS	III	WB, IHC
8	F	24	10	OXC, VPA, CBZ, PHT	RTN	FIAS	I	WB, IHC
9	F	21	5	OXC, PHT, LTG	LTN	FAS/FBTCS	II	WB, IHC
10	M	39	7	CBZ, VPA, CBL	RTN	FIAS	I	WB, IHC
11	F	31	11	CBZ, VPA, TPM	LTN	FAS	II	WB, IHC
12	M	24	13	OXC, VPA, PHT	RTN	FAS/FBTCS	I	WB, IHC
13	F	22	14	CBZ, PHT, VPA	RTN	FIAS	II	WB, IHC
14	F	19	5	OXC, VPA, PHT	RTN	FIAS/FBTCS	I	WB, IHC
15	F	27	8	VPA, PHT, TMP	LTN	FIAS	I	WB, IHC
16	M	41	20	OXC, CBZ, PHT, TMP	RTN	FAS	II	WB, IHC
17	F	31	16	CBZ, PHT, LTG	RTN	FAS/FBTCS	I	WB, IHC
18	M	28	11	VPA, CBZ, PHT	RTN	FIAS	II	WB, IHC
19	F	15	3	OXC, VPA, LTG	LTN	FIAS	I	WB, IHC
20	F	33	6	OXC, CBZ, LTG	LTN	FIAS/FBTCS	I	WB, IHC
21	M	37	15	OXC, CBL, VPA, TMP	LTN	FIAS	II	WB, IHC
22	F	29	12	OXC, VPA, LTG	RTN	FAS/FBTCS	II	WB, IHC

*F* female, *M* male, *AED* antiepileptic drugs, *CBZ* carbamazepine, *VPA* valproic acid, *CLB* clonazepam, *GBP* gabapentin, *LTG* lamotrigine, *OXC* oxcarbazepine, *PB* phenobarbital, *PHT* phenytoin, *TPM* topiramate, *LTN* left temporal neocortex, *RTN* right temporal neocortex, *FIAS* focal impaired awareness seizure, *FAS* focal aware seizures, *FBTCS* focal-to-bilateral tonic-clonic seizure, *PO* postoperative outcome (Engel class), *WB* western blotting, *IHC* immunohistochemistry

**Table 2:** Clinical data from ten control subjects

Case no.	Gender	Age (year)	Etiologic factor	Resected tissue	Pathology	Seizure	Application
1	M	26	TBI	LTN	Normal	None	WB, IHC
2	M	28	TBI	RTN	Normal	None	WB, IHC
3	M	23	TBI	RTN	Normal	None	WB, IHC
4	F	31	TBI	LTN	Normal	None	WB, IHC
5	F	19	TBI	RTN	Normal	None	WB, IHC
6	M	17	TBI	LTN	Normal	None	WB, IHC
7	F	33	TBI	LTN	Normal	None	WB, IHC
8	F	22	TBI	RTN	Normal	None	WB, IHC
9	M	21	TBI	RTN	Normal	None	WB, IHC
10	F	20	TBI	LTN	Normal	None	WB, IHC

*F* female, *M* male, *TBI* traumatic brain injury, *LTN* left temporal neocortex, *RTN* right temporal neocortex, *WB* western blotting, *IHC* immunohistochemistry

### Animals

Adult male C57 BL/6 mice (21–24 g) were purchased from the Daping Hospital Animal Center of the Army Medical University. All mice were housed under climate-controlled conditions on a 12-h light/dark cycle, which was divided into 24 h Zeitgeber time (ZT) units, with the light being turned on at ZT 0 (7 a.m.) and the light being turned off at ZT 12 (7 p.m.). Food and water were available ad libitum. All animal experimental procedures were reviewed and approved by the Internal Animal Care and Use Committee of the Army Medical University. Appropriate measures were taken during experimental procedures to minimize animal suffering and reduce the number of animals used.

### The pilocarpine model

Mice were injected with pilocarpine (280–300 mg/kg, i.p., Sigma-Aldrich, USA) to induce SE [1]. Scopolamine methyl nitrate (5 mg/kg, i.p., Sigma-Aldrich, USA) was injected 30 min prior to pilocarpine administration in order to limit the peripheral muscarinic effects [21]. Seizure severity was rated using the Racine scale [22]. Categories 1 and 2 include facial automatisms, tail stiffening, and wet-dog shakes; category 3, the low-intensity tonic-clonic seizure marked by unilateral forelimb myoclonus in addition to the symptoms above; category 4, the addition of bilateral forelimb myoclonus and rearing; and category 5, bilateral fore- and hindlimb myoclonus and transient loss of postural control. Only those mice that attained categories 4–5 were used. Diazepam (10 mg/kg, i.p.) was administered 2 h after the onset of SE to suppress convulsions. The post-SE supportive care of the animals included body temperature maintenance and rehydration therapy to increase the number of survivors. Injections (i.p.) of sterile 0.9% saline were administered for replenish fluids. Sliced peeled apples were placed in all mouse cages in addition to rodent chow. Spontaneous recurrent seizures (SRSs) were found in mice by monitoring after SE, which confirmed the successful establishment of chronic epilepsy model. Age- and weight-matched control mice received the same treatment with scopolamine methyl nitrate and diazepam, and we used saline instead of pilocarpine.

### Animal experimental design

**Fig. 1:**
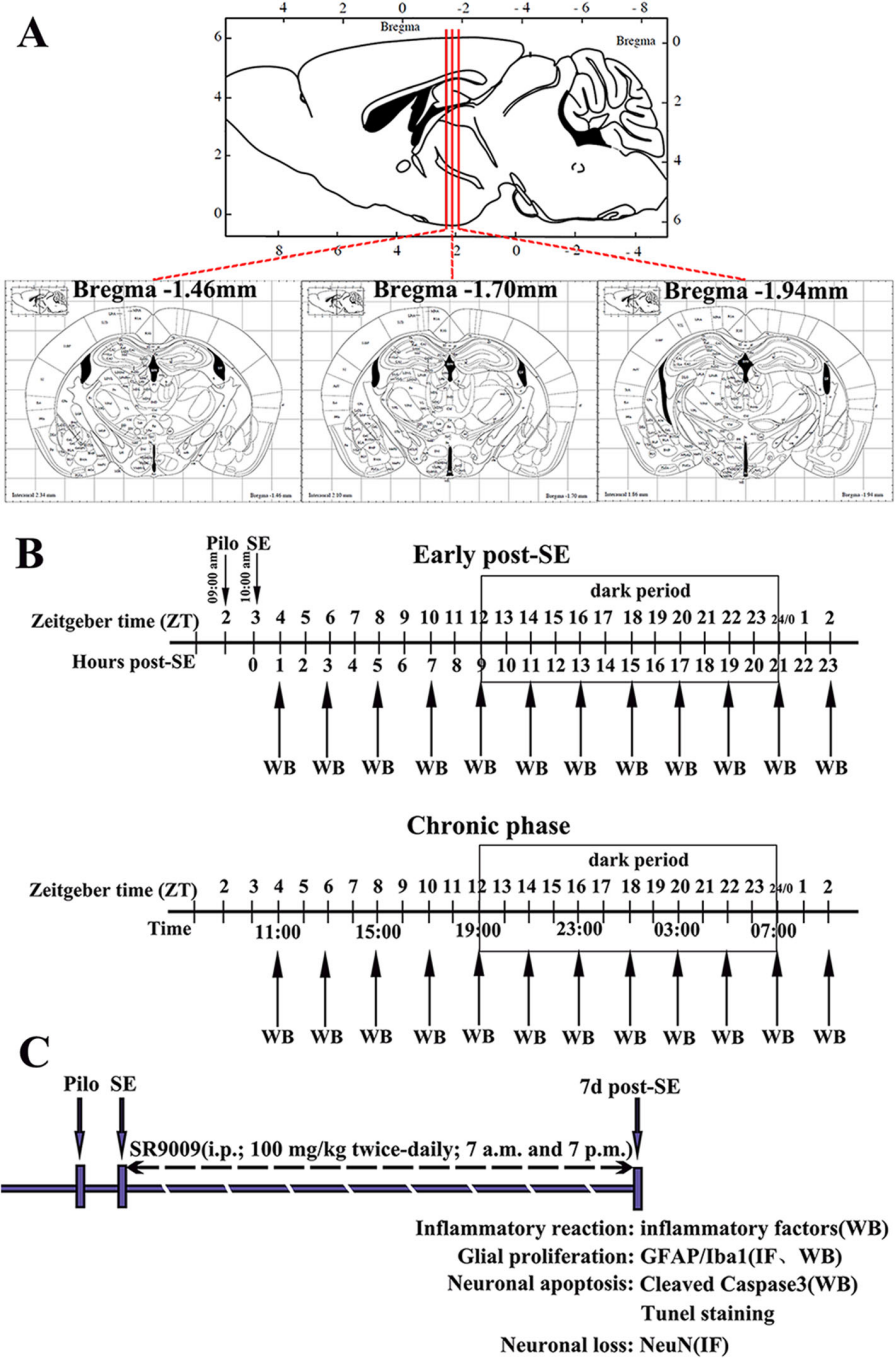
Schematic diagram of the experimental design. **a** Three coordinate positions of coronal sections of mouse brain. **b** Pilocarpine was injected at 9 a.m. (ZT 2). Mice that presented SE until 10 a.m. (ZT 3) were included in the experiments. The expression profiles of Rev-Erbα in the hippocampus and temporal neocortex of mice in the control group, early post-SE group, and chronic epilepsy group were determined by WB. Mice were euthanized every 2 h throughout the 24-h light-dark cycle at ZT 4, 6, 8, 10, 12, 14, 16, 18, 20, 22, 0, and 2 (5 controls and 5 experimental animals at each time point). The box indicates the dark period. **c** Schematic of SR9009 intervention therapy. SR9009 (100 mg/kg) was administered intraperitoneally twice daily (7 a.m. and 7 p.m.) for 7 consecutive days after SE. Then, we explored the effects of SR9009 on the inflammatory reaction, glial proliferation, neuronal apoptosis, and neuronal loss in the hippocampus 7 days after SE. PILO, pilocarpine; SE, status epilepticus; WB, western blotting; IF, immunofluorescence staining

In experiment 1, the expression profiles of Rev-Erbα in the hippocampus and temporal neocortex of mice in the control group (*n* = 60), the early post-SE group (*n* = 60), and the chronic epilepsy group (*n* = 60) were determined by western blotting (Fig. 1b). Mice were euthanized every 2 h over 24 h at ZT 4, 6, 8, 10, 12, 14, 16, 18, 20, 22, 0, and 2 period (five controls and five experimental per time point). These mice were euthanized by decapitation using a guillotine within 20 min of each ZT. For ZT 12, 14, 16, 18, 20, and 22 (dark period points), euthanasia was carried out under dim red light.

In experiment 2, we explored the effects of SR9009 on inflammatory reactions, glial proliferation, neuronal apoptosis, and neuronal loss in the hippocampus 7 days after pilocarpine induced-SE (Fig. 1c). In total, 44 mice were distributed into the following four groups: the vehicle-treated control group, the SR9009-treated control group, the vehicle-treated pilocarpine group, and the SR9009-treated pilocarpine group. SR9009 is a specific brain-permeable small molecule agonist of Rev-Erbα [23–25]. SR9009 (MedChem Express, USA) was dissolved in DMSO at a final concentration of 0.1% DMSO and administered intraperitoneally at a dose of 100 mg/kg twice daily (7 a.m. and 7 p.m.) for 7 consecutive days [15, 16]. The first injection was 1 h after SE. It should be noted that SR9009 had no effect on the expression levels of Rev-Erbα in the control and SE model animals (data not shown).

### Tissue preparation

Tissues from patients were immediately collected at the time of surgery. One set of tissue was immediately frozen in liquid nitrogen and stored at − 80 °C for western blotting. The other samples were fixed in 4% phosphate-buffered paraformaldehyde for 48 h, and the tissues were then embedded in paraffin, sectioned at a thickness of 6 μm for immunohistochemistry and 10 μm for immunofluorescence staining. The bilateral temporal neocortex and hippocampus were dissected from mice for protein extraction and western blotting. For immunofluorescence and TUNEL staining, the mouse brains were obtained after systemic saline and 4% phosphate-buffered paraformaldehyde infusion and then further processed as described above for human tissues.

### Protein extraction and western blotting analysis

A whole protein extraction kit (Beyotime, Jiangsu, China) was used to extract total protein from tissues. Total tissue lysates were centrifuged at 14,000 rpm for 15 min at 4 °C, and the protein concentration in the supernatant was determined using the bicinchoninic acid protein assay (Bio-Rad, Hercules, CA, USA). Equal amounts of protein (60 μg per lane) were separated by sodium dodecyl sulfate-polyacrylamide gel electrophoresis (SDS-PAGE) (5% spacer gel, 80 V, 20–30 min; 8–10% separating gel, 120 V, 50–60 min) and electrophoretically transferred to polyvinylidene fluoride (PVDF) membranes (Millipore, Temecula, CA, USA) using a semidry electroblotting system (Transblot SD; Bio-Rad) (300 mA, 80 min). Then, blotting membranes were incubated at room temperature for 4 h in 5% nonfat dry milk to block nonspecific binding. Next, the membranes were incubated overnight at 4 °C with primary antibodies: rabbit anti-Rev-Erbα (1:1000, Abcam, UK), rabbit anti-NLRP3 (1:1000, Abcam, UK), rabbit anti-ASC (1:1000, Immunoway, USA), rabbit anti-Caspase1 (1:200, Boster, Wuhan, China), rabbit anti-IL-1β (1:1000, Abcam, UK), rabbit anti-IL-18 (1:1500, Invitrogen, USA), rabbit anti-IL-6 (1:1000, Abcam, UK), rabbit anti-TNF-α (1:1000, Abcam, UK), mouse anti-GFAP (1:500, Sigma, USA), rabbit anti-Iba1 (1:800, Wako), rabbit anti-cleaved Caspase3 (1:500, Cell Signaling Technology, Boston, MA), and rabbit anti-GAPDH (1:12000, Abcam, UK). After four rinses in TBST (20 mM Tris-HCl, pH 8.0, 150 mM NaCl, and 0.5% Tween-20), the samples were treated with a horseradish peroxidase-conjugated goat anti-rabbit or goat anti-mouse secondary antibody (1:1000, Zhongshan Golden Bridge Biotechnology, China) for 1 h in a 37 °C incubator. The immunoreactive bands were visualized using enhanced chemiluminescence and were scanned and analyzed with Quantity One software (Bio-Rad Laboratories, Hercules, CA, USA). The optical densities (ODs) of each protein band were calculated relative to the OD of the reference protein, GAPDH.

### Immunohistochemistry (IHC)

Formalin-fixed, paraffin-embedded sections (6 μm thick) were mounted on polylysine-coated slides. Paraffin sections were dewaxed in xylene and rehydrated in a series (100%, 90%, 75%, 50%) of ethanol. Sections were incubated for 30 min in 0.3% hydrogen peroxide in methyl alcohol to inactivate endogenous peroxidase. Antigen retrieval was performed by boiling in a microwave for 10 min in citrate buffer (pH 6.0). The sections were then blocked in 5% bovine serum albumin and 0.3% Triton X-100 for 1 h at room temperature. After the removal of excess serum, the sections were incubated with primary antibody (rabbit anti-Rev-Erbα, 1:100, Abcam, UK) overnight at 4 °C. After washing, the sections were incubated with a HRP-conjugated goat anti-rabbit IgG secondary antibody (Boster, China) for 1 h at 37 °C. Then, the color was developed using 3,3′-diaminobenzidine (DAB; Boster, China) substrate. Finally, the sections were dehydrated, diaphanized, and covered with coverslips. No immunoreactive cells were observed in the negative control experiments, which involved the omission of the primary antibody, preabsorption with a tenfold excess of specific blocking antigen, or incubation with an isotype-matched rabbit polyclonal antibody. Images of Rev-Erbα immunostaining for each clinical surgical specimen section were captured with a × 40 objective (Olympus microscope digital camera system, DP80, Tokyo, Japan). The camera aperture and power and the exposure time of all images were constant. Five nonoverlapping gray matter visual fields and five nonoverlapping white matter visual fields from each section were randomly chosen for further semiquantitative analysis of Rev-Erbα expression (Image-Pro Plus 6.0 software, Media Cybermetrics Inc., USA). For each field, areas of interest were selected to measure the integrated optical density (OD) and area. Moreover, each integrated OD/area value was calculated by subtracting the background integrated OD/area value from the directly measured integrated OD/area value.

### Immunofluorescence staining

For double immunofluorescence staining, paraffin sections from patients were incubated at 4 °C overnight with a rabbit anti-Rev-Erbα primary antibody (1:100, Abcam, UK) combined with mouse anti-NeuN (1:200, Millipore, USA), mouse anti-GFAP (1:500, Sigma, USA), and mouse anti-Iba1 (1:300, Wako) primary antibodies. After rinsing in Tris-buffered saline (TBS) (4 times × 10 min), the sections were incubated with a mixture of secondary antibodies (Cy3-conjugated donkey anti-mouse, 1:400; 488-conjugated donkey anti-rabbit, 1:400; Jackson ImmunoResearch, West Grove, PA, USA) for 1 h at 37 °C. For fluorescence label staining for NeuN, GFAP, and Iba1 in mouse model sections, the three abovementioned primary antibodies and a Cy3-conjugated donkey anti-mouse secondary antibody (1:400, Jackson ImmunoResearch, West Grove, PA, USA) were used. The nuclei were counterstained with 4′,6-diamidino-2-phenylindole (DAPI; Boster, China). The fluorescence signals were acquired using a confocal fluorescence microscope (TCS-SP5, Leica, Nussloch, Germany).

Based on previous reports [26, 27], we evaluated the density of neurons, astrocytes, and microglia in the CA1, CA3, and dentate gyrus (DG) regions of the hippocampus using a modified method. Coronal sections of the brain from similar anatomical positions (Fig. 1a; three coordinate positions: 1.46 mm, 1.70 mm, and 1.94 mm posterior to bregma; three consecutive sections at each position) were collected from each mouse. Three areas from the CA1, CA3, or DG region of each section were randomly selected, three sections from each coordinate position form each mouse, and three mice from each group were measured, that is, 27 areas of the CA1, CA3, or DG region were analyzed for each group. The number of NeuN^+^ neurons, GFAP^+^ astrocyte cell soma, and Iba1^+^ microglia cell soma were counted using ImageJ software (version 1.42 V, NIH, USA). These analyses were conducted in a blinded fashion. The results are expressed as the mean ± SD number of cells per square millimeter.

### TUNEL staining

Pretreated of 6-μm thick paraffin-embedded sections were processed as described for the immunohistochemistry procedure. Apoptosis was determined with the TUNEL kit (Boster, China) according to the manufacturer’s protocol. Section positions were selected as described above (Fig. 1a). Images of TUNEL staining were captured using an Olympus microscope (DP80, Olympus, Tokyo, Japan) with a × 40 objective. Image-Pro Plus 6.0 software was used to count TUNEL-positive cells in each visual field. Three microscopic fields of the CA1 or CA3 region from each section, three sections from each mouse, and three mice from each group were used. All of the mouse sections used for the analysis were viewed in a blinded manner. We averaged the results for each group.

### Statistical analysis

GraphPad Prism 8 (GraphPad Software, La Jolla, CA, USA) was used for statistical analyses. The analysis between the TLE patients and the controls was carried out using Student’s *t* test. Two-way ANOVA followed by post hoc Bonferroni’s test was used to compare the groups (control and experimental) throughout the 24-h period by western blotting analysis. One-way ANOVA analysis followed by post hoc Bonferroni’s test was used to determine the differences among multiple groups. The data are expressed as the mean ± SD. Significance was accepted at *p* < 0.05.

## Results

### Clinical characteristics

An examination of sex and age revealed no difference between the subjects in the control and TLE groups. The control group contained 5 female and 5 male individuals with a mean age of 24.00 ± 1.680 years (range, 17–33 years). The mean age of the intractable TLE patients was 27.09 ± 1.475 years (range, 15–41 years), with 10 males and 12 females.

### Decreased expression of Rev-Erbα in the temporal neocortex of TLE patients

**Fig. 2:**
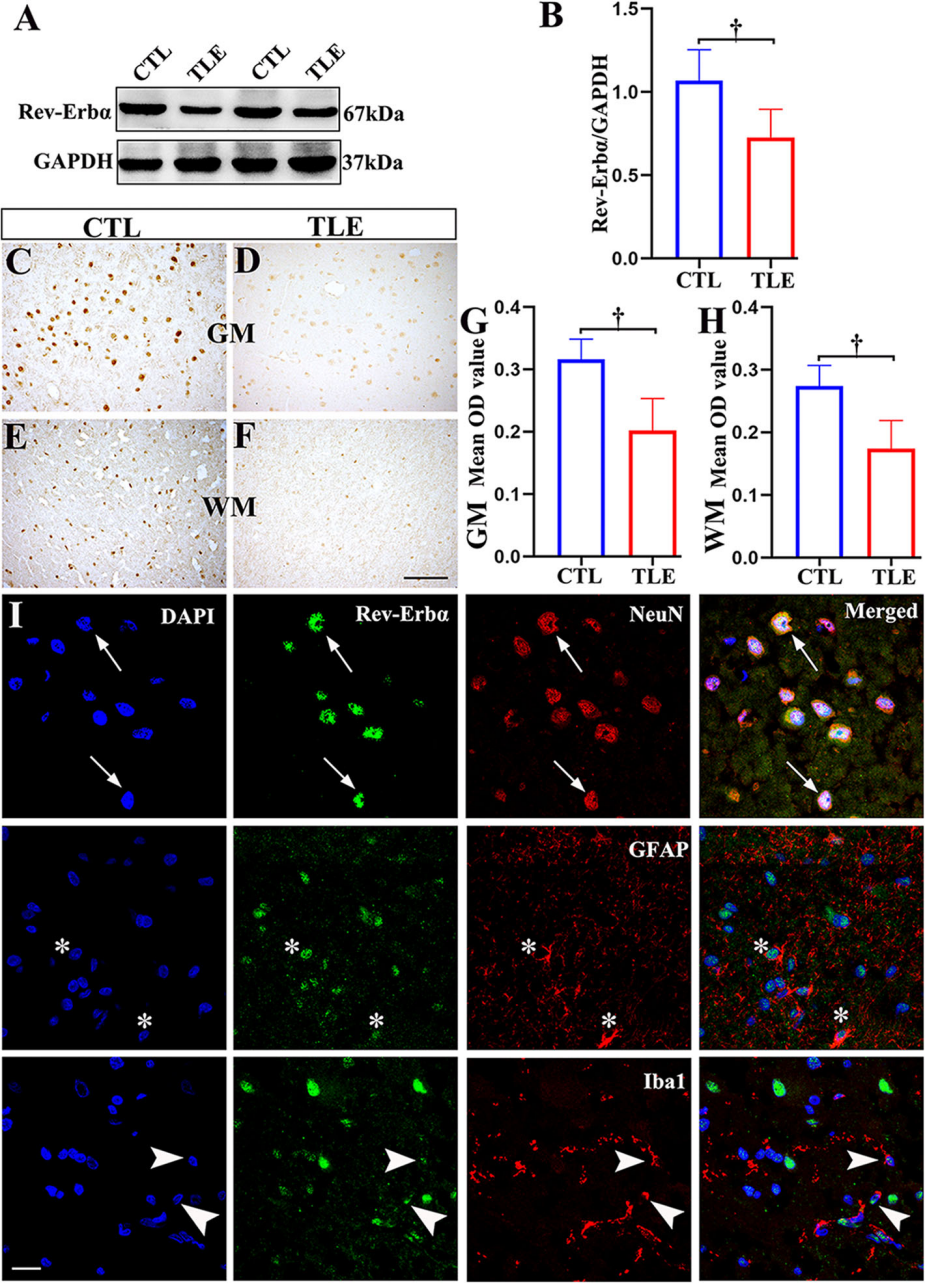
The expression patterns of Rev-Erbα in the temporal neocortex of patients with TLE and controls (CTLs). **a** The Rev-Erbα protein level was detected by western blotting in the temporal neocortex of TLE patients (*n* = 22) and controls (*n* = 10). **b** Densitometric analysis indicated that protein immunoreactivity for Rev-Erbα was decreased in TLE patients. In controls (*n* = 10), Rev-Erbα displayed moderate to strong IR in neurons within the gray matter (GM; **c**) and in glial cells within the white matter (WM; **e**). Rev-Erbα showed weak IR in corresponding regions in the TLE group (*n* = 22; **d**, **f**). The mean OD of Rev-Erbα in the GM (**g**) and WM (**h**) was significantly reduced in the TLE group compared with the control group. **i** Double-labeled immunofluorescence staining showed that Rev-Erbα was coexpressed with NeuN in neurons (arrows). Rev-Erbα was colocalized with GFAP in astrocytes (asterisks); Rev-Erbα and Iba1 were also colocalized (arrowheads). The data are expressed as the mean ± SD. ^†^*p* < 0.001. Scale bar = 50 μm for **c**–**f**, **i**

We determined the protein expression levels of Rev-Erbα in the temporal neocortex specimens from TLE patients (*n* = 22) and controls (*n* = 10) by western blotting. Rev-Erbα and GAPDH immunoreactive bands were observed at approximately 67 kDa and 37 kDa (Fig. 2a), respectively. Compared with specimens from the controls, the temporal neocortex specimens from TLE patients had significantly lower levels of Rev-Erbα protein (*p* < 0.001; Fig. 2b).

### Distribution and comparison of Rev-Erbα protein immunoreactivity (IR) in controls and TLE patients

Next, we analyzed the expression patterns of Rev-Erbα using IHC and immunofluorescence labeling staining. In controls (*n* = 10), Rev-Erbα displayed moderate to strong IR in neurons within the gray matter (GM; Fig. 2c) and glial cells within the white matter (WM; Fig. 2e). However, in the TLE patient group, Rev-Erbα showed weak IR in corresponding regions (Fig. 2d, f; *n* = 22). The optical density (OD) analysis revealed that the mean OD of Rev-Erbα in the GM and WM was significantly reduced in the TLE group compared with the control group. (Fig. 2g, h; *p* < 0.001). Subsequently, double-labeled immunofluorescence experiments (Fig. 2i) demonstrated that Rev-Erbα was colocalized with the neuronal marker NeuN and the astrocyte marker GFAP. In addition, Rev-Erbα IR was detectable in Iba1-positive microglia.

### Temporal profiling of Rev-Erbα protein levels in the pilocarpine model of TLE

AEDs may have an effect on human Rev-Erbα expression. Additionally, Rev-Erbα is a circadian regulator that may present 24-h cyclic patterns in some brain regions [28, 29], and although each operation was performed between 10 a.m. and 1 p.m., the timing of the surgical removal of the temporal neocortex from epileptic patients and control patients may not have been entirely consistent. Thus, we assessed the temporal changes in Rev-Erbα protein levels in the hippocampus and temporal neocortex of control mice and mice subjected to SE (early post-SE and the chronic phases), which were sacrificed every 2 h over a 24 h timescale.

**Fig. 3:**
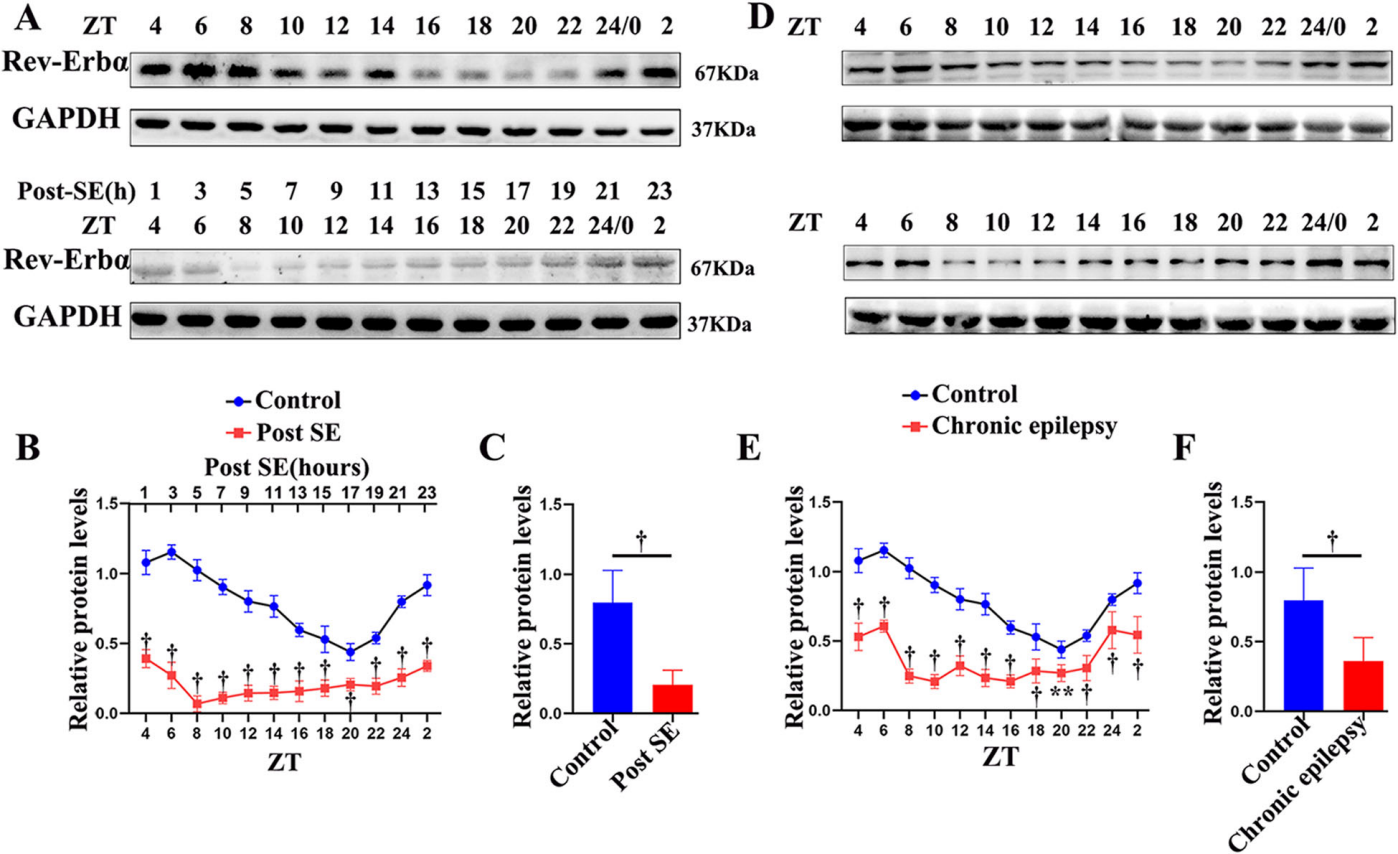
Relative protein expression of Rev-Erbα in the hippocampus of mice in the early post-SE (**a**–**c**) and chronic phases (**d**–**f**). **a**, **d** A representative western blot image of Rev-Erbα (67 kDa) and corresponding GAPDH (37 kDa) expression in the hippocampus. Lanes 1–12 represent different time points (ZT 4, 6, 8, 10, 12, 14, 16, 18, 20, 22, 0, and 2). **b**, **e** A comparison of the intensity ratio of Rev-Erbα expression between the control and the experimental mice at different time points in the hippocampus in the early post-SE (**b**) and chronic phases (**e**). **c**, **f** The analysis of the mean protein expression (the mean expression at all ZTs) demonstrated that Rev-Erbα was significantly reduced in both the early post-SE (**c**) and chronic phases (**f**). *n* = 5 for each point. **p < 0.01, ^†^*p* < 0.001

In the hippocampus, a pronounced diurnal rhythm of Rev-Erbα expression was observed in the control group. The time points of the peak (ZT 6; light period) and nadir (ZT 20; dark period) expression in the control group are shown in Fig. 3. However, the oscillatory rhythm of Rev-Erbα was lost in the early post-SE phase. The Rev-Erbα protein expression in the mouse hippocampus during the early post-SE phase was decreased in both the light and dark periods of the 24-h cycle when compared with that in the controls (Fig. 3a, b; *p* < 0.001). The expression of Rev-Erbα dropped to its lowest level after 5 h of SE (ZT 8). Remarkably, 60 days after SE induction (in the chronic phase), the expression of Rev-Erbα was still not rhythmic and was maintained at a relatively low level (Fig. 3d, e; *p* < 0.001 or *p* < 0.01). Furthermore, analysis of the mean protein expression (the mean of the levels at all ZTs) demonstrated that Rev-Erbα was significantly reduced in both the early post-SE and chronic phases (Fig. 3c, f; *p* < 0.001).

**Fig. 4:**
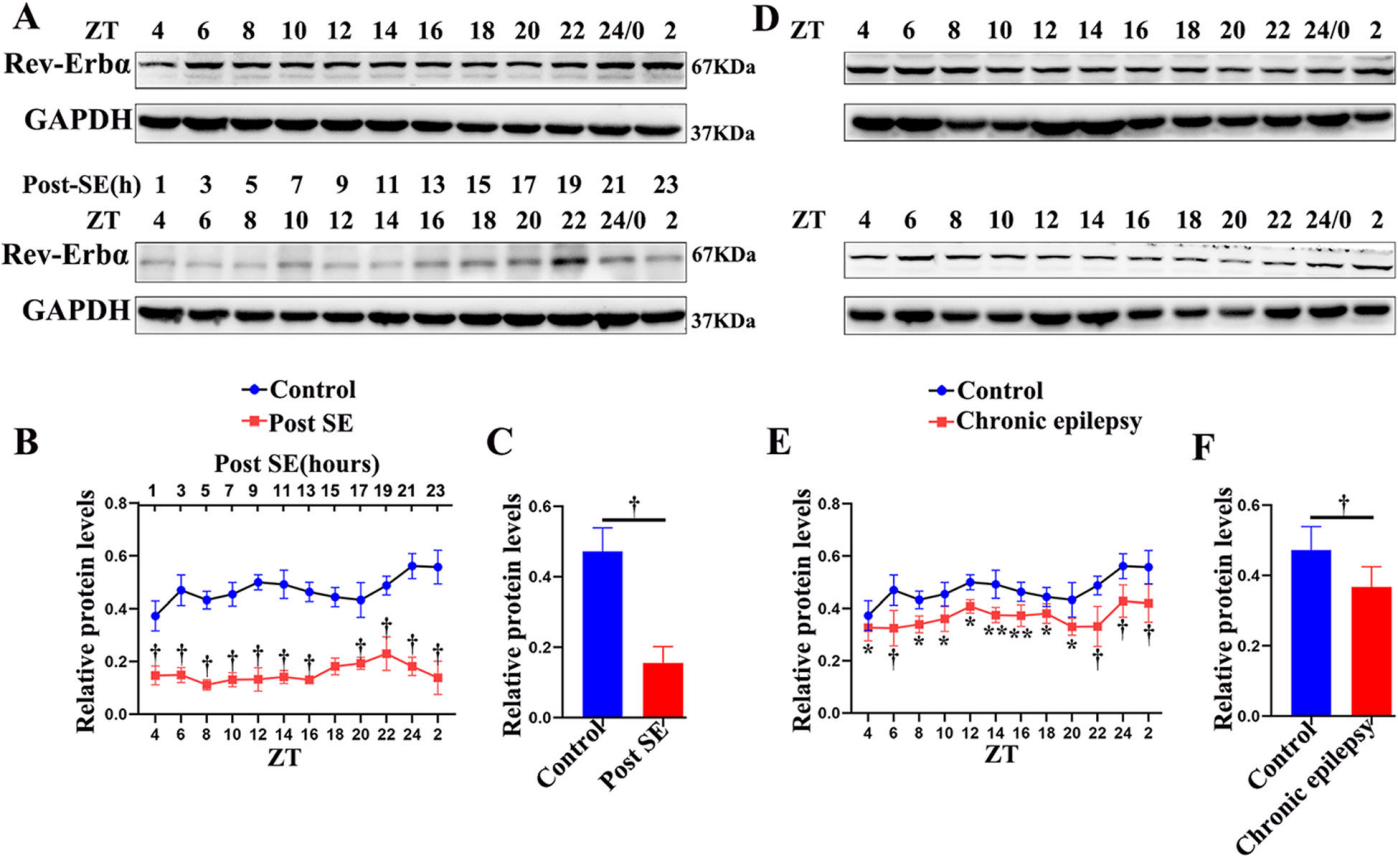
Western blotting analysis of Rev-Erbα in the temporal neocortex of mice in the early post-SE (**a**–**c**) and chronic phases (**d**–**f**). The Rev-Erbα protein was detected as an approximately 67 kDa band. The analysis shows the protein expression of Rev-Erbα in the early post-SE group (**b**, **c**) and chronic phase group (**e**, **f**) compared to that in the controls throughout the circadian cycle. *n* = 5 for each point. The data are expressed as the mean ± SD. *p < 0.05, **p < 0.01, ^†^*p* < 0.001

As depicted in Fig. 4, Rev-Erbα expression did not present a 24-h rhythm in the temporal neocortex under control conditions. In addition, the experimental mice (early post-SE and chronic phases) had lower levels of Rev-Erbα protein both during the light and during the darkness (Fig. 3a, b, d, e; *p* < 0.001 or *p* < 0.05). Compared with that in the control group, the mean protein expression of Rev-Erbα decreased significantly in both early post-SE and chronic conditions (Fig. 4c, f; *p* < 0.001). These animal experimental data suggest that Rev-Erbα expression decreases throughout the epileptogenesis process of TLE.

### SR9009 administration inhibited NLRP3 inflammasome activation and inflammatory cytokine production after SE

**Fig. 5:**
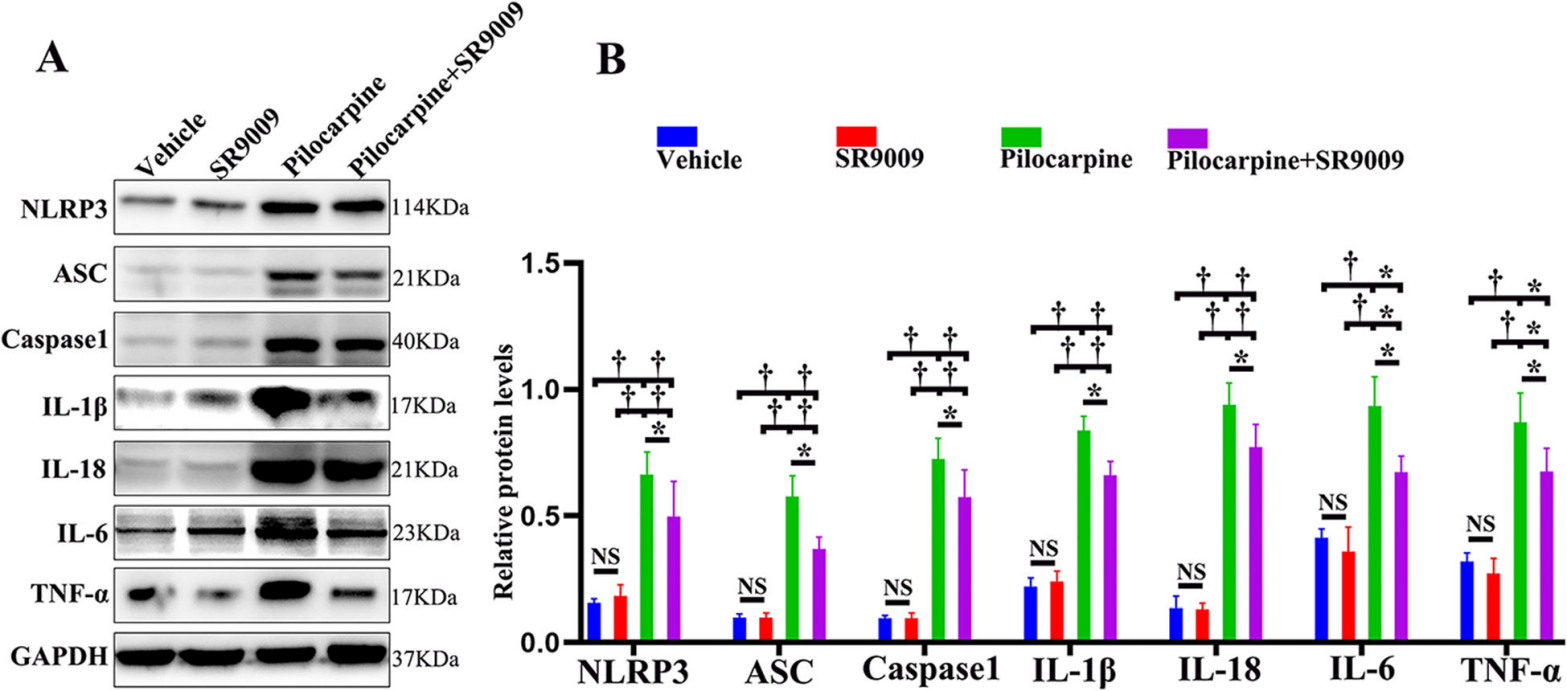
The application of SR9009 for 7 days reduced the expression of NLRP3 inflammasome-related proteins and inflammatory cytokines in the hippocampus after SE. **a** The expression of NLRP3, ASC, Caspase1, IL-1β, IL-18, IL-6, and TNF-α was detected by western blotting assay. **b** Densitometric analysis results of NLRP3, ASC, Caspase1, IL-1β, IL-18, IL-6, and TNF-α in the four groups. *n* = 5 for each group. The data presented as the mean ± SD. NS, not significant, *p < 0.05, ^†^*p* < 0.001

The NLRP3 inflammasome is a cytosolic protein component that interacts with the inflammasome-adaptor protein ASC, leading to caspase1 activation [30]. Activated caspase-1 catalyzes the maturation of the proinflammatory cytokines IL-1β and IL-18 [31]. It has been suggested that the release of inflammatory factors strongly promotes epileptogenesis [32]. Recent studies have highlighted the emerging role of Rev-Erbα in the inflammatory cascade [17]. To determine the influences of SR9009 on NLRP3 inflammasome activation and the subsequent secretion of inflammatory cytokines after SE, we estimated the levels of NLRP3, ASC, Caspase1, IL-1β, IL-18, IL-6, and TNF-α in the hippocampus by western blotting assay. SR9009 did not affect the activation of the NLRP3 inflammasome or the production of inflammatory cytokines under normal physiological conditions (vehicle-treated control group vs. SR9009-treated control group; *p* > 0.05). The concentrations of NLRP3, ASC, Caspase1, IL-1β, IL-18, IL-6, and TNF-α were significantly increased in the vehicle-treated pilocarpine group and the SR9009-treated pilocarpine group compared to the vehicle-treated control group and the SR9009-treated control group, respectively (Fig. 5a, b; *p* < 0.001 or *p* < 0.05). The protein levels of NLRP3, ASC, Caspase1, IL-1β, IL-18, IL-6, and TNF-α were significantly decreased in the SR9009-treated pilocarpine group compared with the vehicle-treated pilocarpine group (Fig. 5a, b; *p* < 0.05). These results suggest that the continuous use of SR9009 for 7 days can reduce the inflammatory reaction in the hippocampus after SE.

### SR9009 application attenuated astrocytosis and microgliosis in the pilocarpine-induced SE model

**Fig. 6:**
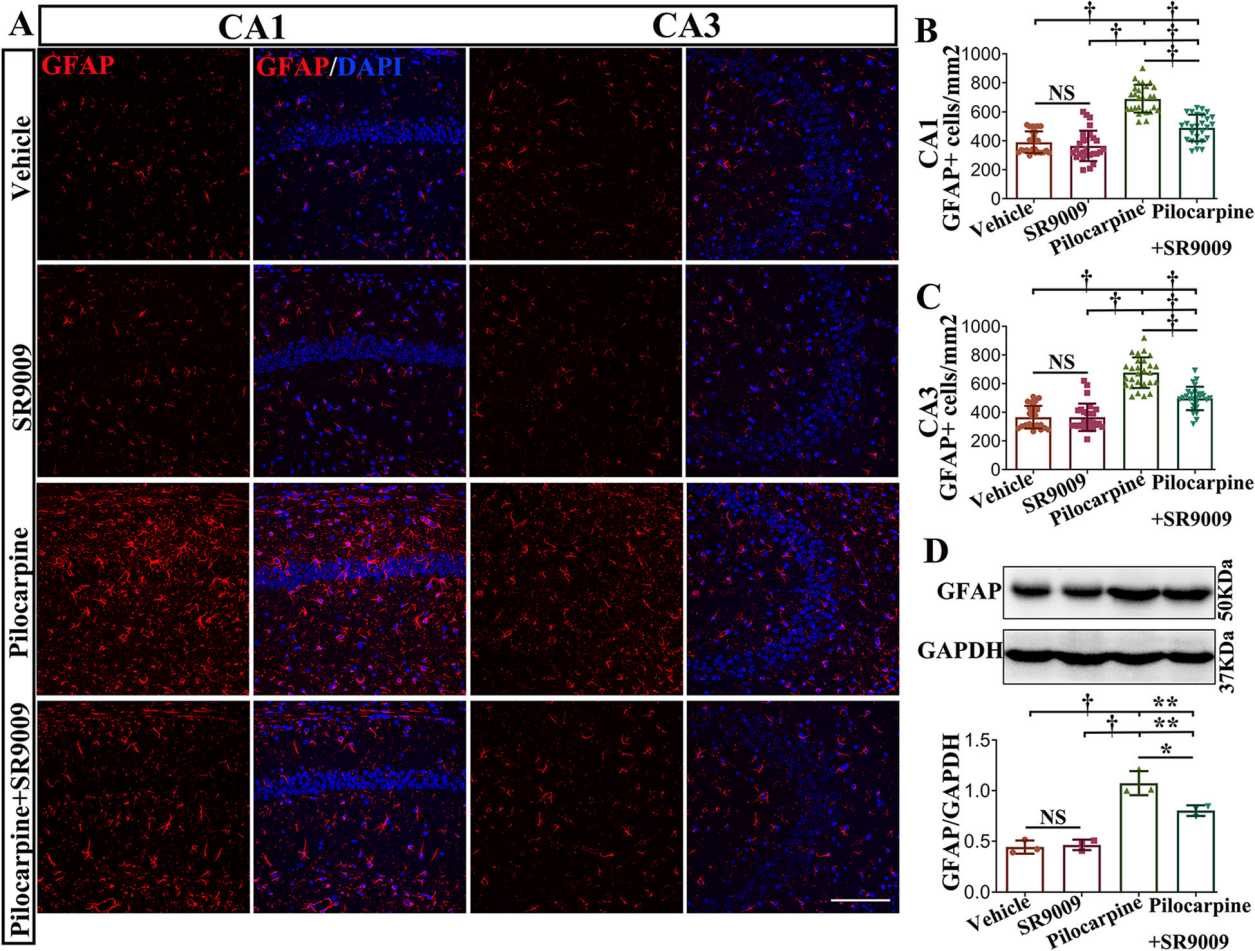
SR9009 application for 7 consecutive days attenuated astrocyte activation in the pilocarpine-induced SE model. **a** GFAP immunofluorescence staining in the CA1 and CA3 regions of the hippocampus in vehicle-treated control mice, SR9009-treated control mice, vehicle-treated pilocarpine mice, and SR9009-treated pilocarpine mice. **b**, **c** The level of GFAP-positive cells in the CA1 and CA3 regions was decreased in SR9009-treated pilocarpine mice compared to the vehicle-treated pilocarpine mice. **d** Western blotting analysis showed that SR9009 significantly reduced the protein level of GFAP in the hippocampus after SE. *n* = 3 for each group. The data presented as the mean ± SD. NS, not significant, *p < 0.05, **p < 0.01, ^†^*p* < 0.001. Scale bar = 50 μm

The proliferation and activation of astrocytes and microglia are regarded as typical pathological processes of TLE [33]. To evaluate the effect of SR9009 administration on glial activation in the hippocampus, immunofluorescence staining against GFAP was conducted on coronal brain sections on day 7 post-SE (Fig. 6a). The number of GFAP-positive cells in the hippocampal CA1 and CA3 regions was quantified and compared in different experimental groups (Fig. 6b, c). Our data demonstrated that there was no significant difference between vehicle-treated control mice and SR9009-treated control mice in either region of the hippocampus (*p* > 0.05). A significant increase in the number of GFAP-expressing cells in the CA1 and CA3 regions was found in vehicle-treated pilocarpine mice and SR9009-treated pilocarpine mice compared to vehicle-treated control mice and SR9009-treated control mice (*p* < 0.001). Additionally, the number of GFAP-positive cells in CA1 and CA3 regions was decreased in SR9009-treated pilocarpine mice compared to vehicle-treated pilocarpine mice (*p* < 0.001). Next, we evaluated the protein level of GFAP (50 kDa) in the hippocampus of the four groups by western blotting (Fig. 6d). Pilocarpine strongly increased GFAP expression in the hippocampus (vehicle-treated pilocarpine mice vs. vehicle-treated control mice and SR9009-treated control mice; *p* < 0.001). In comparison with the vehicle-treated pilocarpine mice, the SR9009-treated pilocarpine mice had lower GFAP levels (*p* < 0.05), but they had higher GFAP levels than the vehicle-treated control mice and SR9009-treated control mice (*p* < 0.01).

**Fig. 7:**
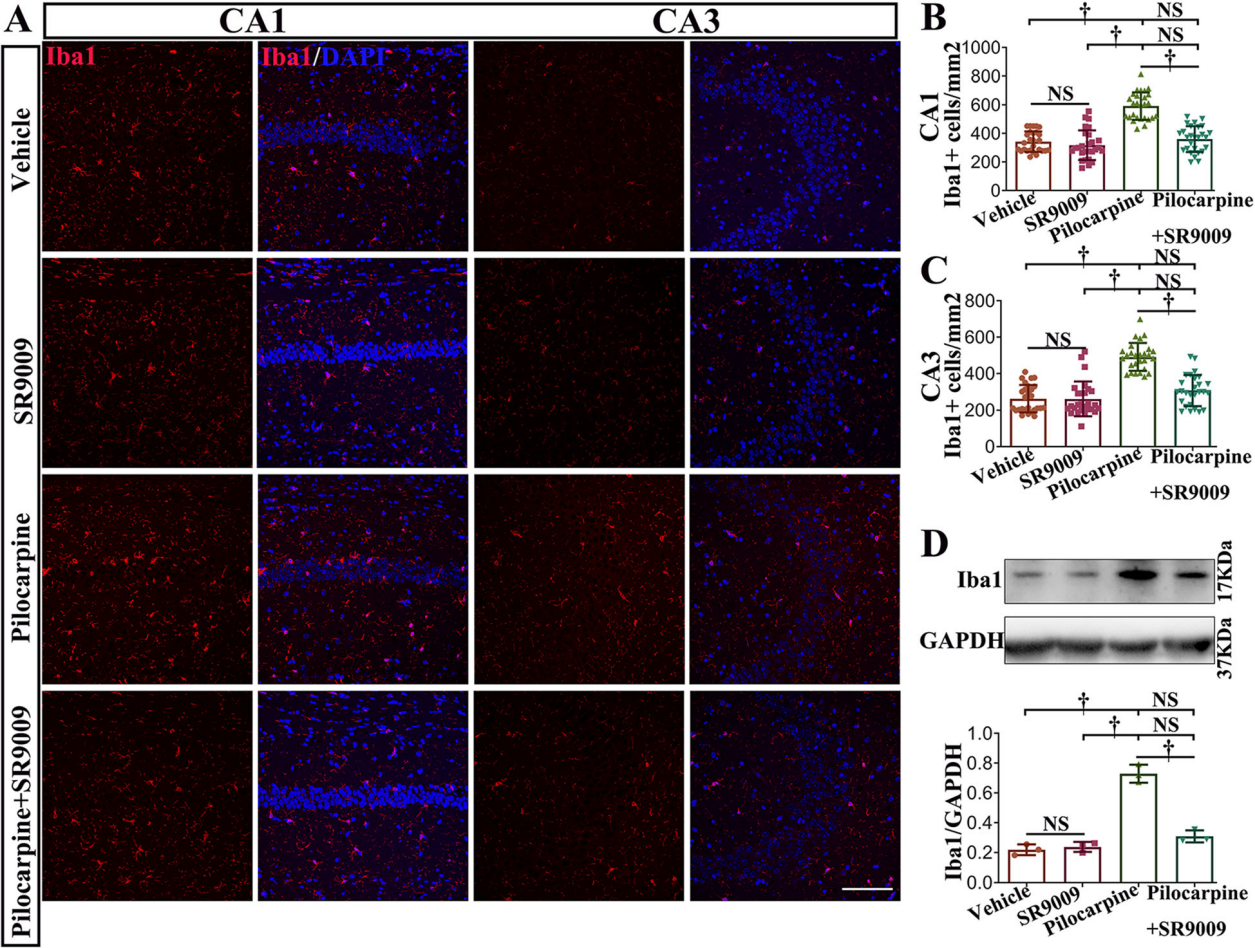
SR9009 application attenuated microglial activation in the pilocarpine-induced SE model. **a** Iba1 immunofluorescence staining in the CA1 and CA3 regions of the hippocampus in the four groups. **b**, **c** The number of microglia was drastically decreased in SR9009-treated pilocarpine mice compared with vehicle-treated pilocarpine mice. **d** Western blotting analysis demonstrated that SR9009 significantly reduced the protein level of Iba1 in the hippocampus after SE. *n* = 3 for each group. The data presented as the mean ± SD. NS, not significant, ^†^*p* < 0.001. Scale bar = 50 μm

Additionally, pilocarpine treatment significantly increased the number of Iba1-positive cells in the CA1 and CA3 regions of the hippocampus (vehicle-treated pilocarpine mice vs. SR9009-treated control mice or vehicle-treated control mice; Fig. 7a, b; *p* < 0.001). The number of Iba1-positive cells was drastically decreased in SR9009-treated pilocarpine mice compared with vehicle-treated pilocarpine mice (*p* < 0.001). There was no difference between control mice and SR9009-treated pilocarpine mice in either region of the hippocampus (vehicle-treated control mice vs. SR9009-treated pilocarpine mice, *p* > 0.05; SR9009-treated control mice vs. SR9009-treated pilocarpine mice, *p* > 0.05). The number of Iba1-positive cells in the SR9009-treated control mice was approximately equal to that in the vehicle-treated control mice (*p* > 0.05). To further confirm the immunofluorescence staining results, the effect of SR9009 on the protein level of Iba1 (17 kDa) in the hippocampus was assessed by western blotting (Fig. 7d). SR9009 did not affect the expression of Iba1 in the hippocampus under normal conditions (*p* > 0.05). The Iba1 level was upregulated in the vehicle-treated pilocarpine mice compared to the vehicle-treated control mice and the SR9009-treated control mice (*p* < 0.001). It is worth noting that the application of SR9009 for 7 consecutive days after SE induction significantly reduced the protein level of Iba1 in hippocampus (*p* < 0.001). No difference in Iba1 protein level between the control mice (vehicle-treated control mice or SR9009-treated control mice) and the SR9009-treated pilocarpine mice was detected (*p* > 0.05). These studies indicate that SR9009 effectively ameliorated the levels of astrocytosis and microgliosis in the early stage post-SE.

### Treatment with SR9009 decreases apoptosis and the loss of hippocampal neurons in SE

**Fig. 8:**
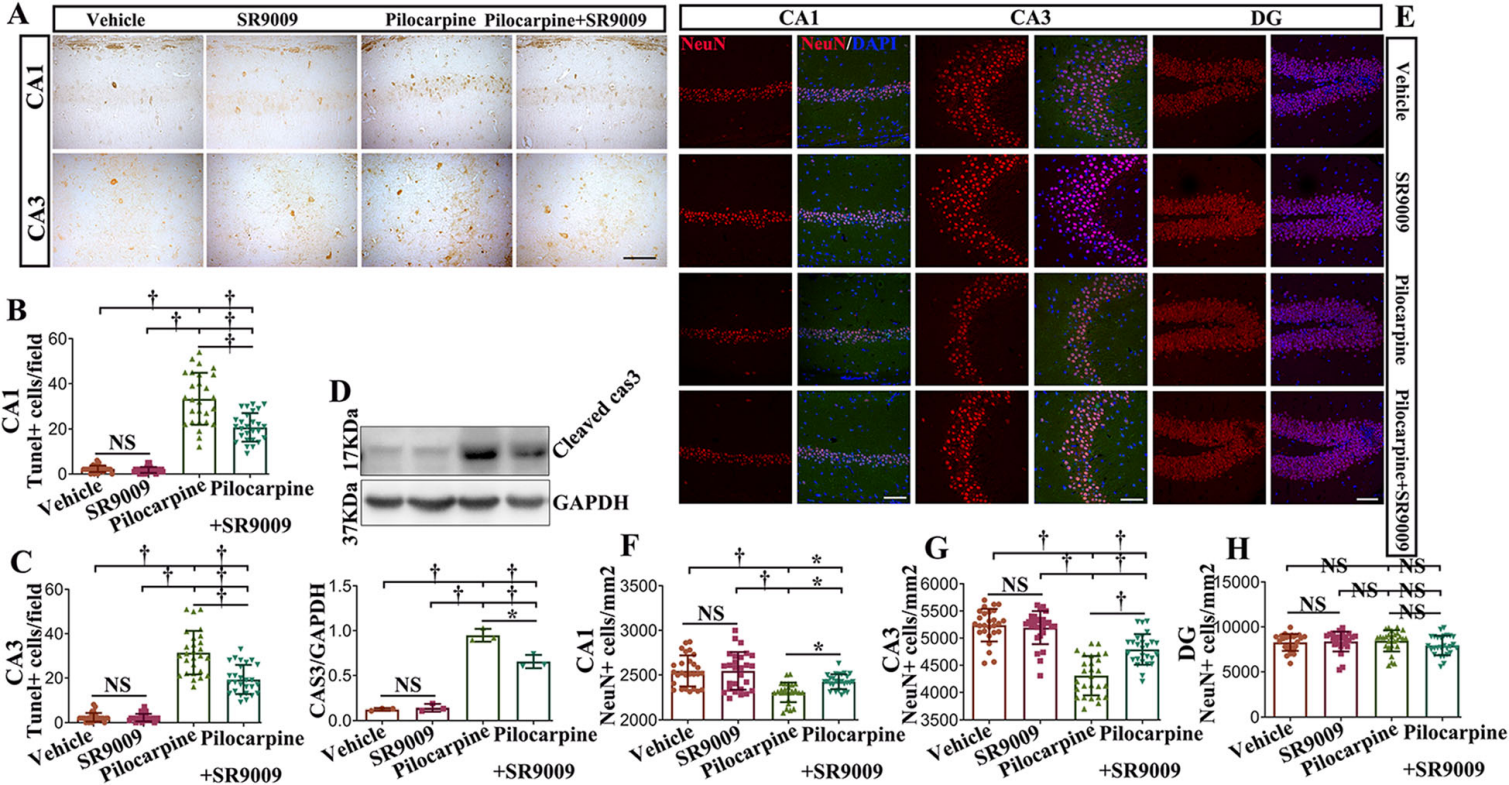
Treatment with SR9009 decreases apoptosis and the loss of hippocampal neurons in the SE. **a** TUNEL-positive cells in the CA1 and CA3 regions of the hippocampus of the four groups in mice are displayed. **b**, **c** The number of TUNEL -positive cells in the CA1 and CA3 regions were calculated. Treatment with SR9009 after SE resulted in a diminished number of TUNEL-positive cells in the CA1 and CA3 regions. **d**, **e** A representative immunoblot showing protein immunoreactivity for cleaved caspase-3 (17 kDa) in the hippocampus of the four groups. A significant decrease in the cleaved-caspase3 level was observed in the SR9009-treated pilocarpine group compared to the vehicle-treated pilocarpine group. **e** NeuN immunofluorescence staining in the CA1, CA3, and DG regions of the hippocampus in the four groups. **f**, **g** The number of NeuN-positive cells in the SR9009-treated pilocarpine group was significantly higher than that in the vehicle-treated pilocarpine group. **h** SR9009 did not affect the number of NeuN-positive cells in the DG after SE. *n* = 3 for each group. The data presented as the mean ± SD. NS, not significant, *p < 0.05, ^†^*p* < 0.001. Scale bar = 50 μm (**a**, **e**)

First, alterations in neuronal apoptosis in the hippocampus were characterized using TUNEL staining (Fig. 8a–c). No significant difference between the two control groups was presented. As expected, SE induced a marked increase in the number of TUNEL-positive cells in the CA1 and CA3 regions compared to that in the vehicle-treated control and SR9009-treated control groups (*p* < 0.001). Treatment with SR9009 after SE resulted in a diminished number of TUNEL-positive cells in the CA1 and CA3 regions (SR9009-treated pilocarpine group vs. vehicle-treated pilocarpine group; *p* < 0.001).

Then, we detected the expression of cleaved-caspase3 (17 kDa) in the hippocampus to determine the apoptotic status of the four groups (Fig. 8d). The level of cleaved caspase3 was increased in the hippocampus of the SR9009-treated pilocarpine group and the vehicle-treated pilocarpine group (*p* < 0.001). Interestingly, a significant decrease in cleaved caspase3 levels was observed in the SR9009-treated pilocarpine group compared to the vehicle-treated pilocarpine group (*p* < 0.001).

To determine the effect of SR9009 on the density of neurons in the hippocampal CA1 and CA3 regions and dentate gyrus (DG), immunofluorescence labeling staining for NeuN was performed on day 7 post-SE (Fig. 8e–h). No differences in the number of NeuN-positive cells were detected in the hippocampal CA1 region, hippocampal CA3 region or DG between the vehicle-treated control and SR9009-treated control groups (*p* > 0.05). We observed that the number of NeuN-positive cells was decreased in the CA1 and CA3 regions of the hippocampus in the vehicle-treated pilocarpine group and the SR9009-treated pilocarpine group compared to that in the two control groups (*p* < 0.05 or *p* < 0.001). Notably, the number of NeuN-positive cells in the SR9009-treated pilocarpine group was significantly higher than the vehicle-treated pilocarpine group (CA1, *p* < 0.05; CA3, *p* < 0.001). SR9009 did not affect the number of NeuN-positive cells in the DG after SE (*p* > 0.05). Collectively, these data demonstrate that SR9009 exerts neuroprotection in the hippocampus in the pilocarpine-induced SE model.

## Discussion

In this study, we found for the first time that Rev-Erbα was downregulated in the epileptogenic foci of TLE patients and mainly localized in neurons, astrocytes, and microglia. The expression of Rev-Erbα was decreased in the hippocampus and temporal neocortex of mice induced by pilocarpine in the early post-SE and chronic phases. It is noteworthy that the expression of Rev-Erbα in the normal hippocampus showed a 24-h rhythm, that the rhythm was disturbed in the early phase after SE, and that this disturbance lasted to the chronic phase. Our further results showed that treatment with SR9009 reduced inflammation and neuronal injury related to SE. These findings support a functional role for Rev-Erbα in TLE and a potentially new therapeutic target for disease modification in TLE.

### Rev-Erbα and neuroinflammation

Clinical observations and data from animal studies have proposed that neuroinflammation is a common factor contributing to, or predisposing one to, the occurrence of seizures in various kinds of epilepsy, especially TLE [34, 35]. For instance, some inflammatory cytokines (IL-1β, IL-6, TNF-α, etc.) synthesized and released by astrocytes and microglia have been reported to significantly contribute to the mechanisms of seizure generation and epileptogenesis [32, 36]. Recently, the NLRP3 inflammasome, a key constituent of the inflammasome complex, has been implicated in the sterile inflammatory response via the processing of caspase-1, IL-18, and IL-1β in the setting of epilepsy and some other neurological diseases [37–39].

Rev-Erbα has been implicated in the regulation of immune activation and cytokine release in various tissues and organs [40, 41]. In studies of NR1D1^−/−^ mice and human macrophages involving the pharmacological activation of Rev-Erbα, researchers have found that Rev-Erbα regulates the expression of NLRP3 and the secretion of cytokines by macrophages [24]. Indeed, recent in vivo and in vitro experiments have suggested Rev-Erbα is an important negative regulator of neuroinflammation [16]. On the one hand, the loss of Rev-Erbα promotes proinflammatory microglial activation, microgliosis, and astrocyte activation in the hippocampus [16]. On the other hand, treatment with Rev-Erbα agonists suppresses inflammatory gene expression in vivo and in cultured glial cells [16–18]. However, little is known about the role of Rev-Erbα in TLE. In the current study, immunohistochemical assays clarified the characteristically low level of expression of Rev-Erbα in glial cells in the epileptic foci of TLE patients. Double labeling experiments delineated that Rev-Erbα colocalized with GFAP and Iba1 in reactive astrocytes and microglia, respectively. Furthermore, SR9009 application for 7 days was effective for suppressing SE-induced inflammation in the hippocampus. This was evinced mainly through reduced protein concentrations of NLRP3, ASC, Caspase1, IL-1β, IL-18, IL-6, TNF-α, GFAP, and Iba1 and diminished numbers of activated astrocytes and Iba1-positive cells in the hippocampus. Hence, it is tempting to propose that the downregulation of Rev-Erbα in the TLE epileptic zone may be involved in the pathophysiology of TLE through the modulation of neuroinflammation. The application of SR9009 after SE may partially alleviate neuroinflammation. It should be noted that both activated resident microglia and infiltrated monocytes contribute to the expansion of Iba1-positive population following pilocarpine-induced SE [42]. Therefore, further investigation is needed to clarify the inhibitory effect of SR9009 on activated resident microglia and infiltrated monocytes.

### Rev-Erbα and neuronal apoptosis/loss

Several studies have demonstrated that Rev-Erbα and neuronal apoptosis are inextricably linked. For example, some investigators have found a significant increase in apoptosis of internal granule cell layer neurons in the cerebellum of NR1D1 mutant mice [19]. Additionally, dopaminergic neuronal loss in the substantia nigra pars compacta (SNpc) induced by 6-hydroxydopamine is accelerated and intensified in Rev-Erbα knockout mice [20]. These studies suggest that Rev-Erbα deficiency may directly or mediately cause neuronal damage in some areas of the brain. In our research, Rev-Erbα was downregulated in surgical samples from intractable TLE patients. We also observed a decrease in Rev-Erbα in the hippocampus and temporal neocortex of mice in both the early and chronic phases after SE. The results of IHC demonstrated that Rev-Erbα was downregulated in the neurons of TLE patients. Importantly, SR9009 application for 7 consecutive days after SE produced a neuroprotective effect in the CA1 and CA3 regions of the hippocampus. It is well recognized that the hippocampus is the region that is most prone to neuronal apoptosis and loss caused by recurrent epileptic seizures, especially initial SE [33, 43, 44]. Accordingly, it is reasonable to presume that the low concentration of Rev-Erbα in epileptic tissue in TLE may aggravate neuronal apoptosis and loss after epileptic seizures. However, it is worth pointing out that a persistent neuroinflammatory response in the epileptic brain may result in neuronal dysfunction, neuronal cell damage, and death [45, 46]. Interestingly, in a primary neuron and glia coculture model, Rev-Erbα-deficient primary glial cultures exacerbate oxidative damage in cultured neurons, which may be related to the increased inflammatory response of Rev-Erbα-deficient glial cells [16]. Therefore, the next step is to elaborate on the specific mechanisms by which Rev-Erbα reduction leads to neuronal apoptosis and loss in TLE.

Our study also has some limitations. Hippocampal lesions are a characteristic pathological change in TLE patients. Unfortunately, there were no normal hippocampal samples obtained from autopsy or by other means during our study period because of ethical principles. The sample size of each group, which is marginally acceptable, may limit the generalizability of our results. In addition, to avoid the influence of traumatic factors on Rev-Erbα expression, we also examined the expression of Rev-Erbα in direct traumatic tissues and distant tissues within 4 h after traumatic brain injury and found no difference between them, which further verified the appropriateness of our control samples (data not shown).

## Conclusions

In conclusion, low expression of Rev-Erbα in the epileptic lesions may determine the intensity of the inflammatory reaction and the fate of neurons, while early activation of Rev-Erbα may have some therapeutic potential.

## Data Availability

The datasets used and/or analyzed during the current study are available from the corresponding author on reasonable request.

## Abbreviations

AEDs: Antiepileptic drugs
CNS: Central nervous system
DMSO: Dimethyl sulfoxide
EEG: Electroencephalogram
GAPDH: Glyceraldehyde-3-phosphate dehydrogenase
GFAP: Glial fibrillary acidic protein
HRP: Horseradish peroxidase
Iba1: Ionized calcium-binding adapter molecule 1
IHC: Immunohistochemistry
ILAE: International League Against Epilepsy
IR: Immunoreactivity
LPS: Lipopolysaccharide
SE: Status epilepticus
SRSs: Spontaneous recurrent seizures
TLE: Temporal lobe epilepsy
TNF: Tumor necrosis factor
TUNEL: Terminal deoxynucleotidyl transferase dUTP nick end labeling
ZT: Zeitgeber time
